# Low Doses of Psilocybin and Ketamine Enhance Motivation and Attention in Poor Performing Rats: Evidence for an Antidepressant Property

**DOI:** 10.3389/fphar.2021.640241

**Published:** 2021-02-26

**Authors:** Guy A. Higgins, Nicole K. Carroll, Matt Brown, Cam MacMillan, Leo B. Silenieks, Sandy Thevarkunnel, Julia Izhakova, Lilia Magomedova, Ines DeLannoy, Edward M. Sellers

**Affiliations:** ^1^ InterVivo Solutions Inc., Fergus, ON, Canada; ^2^ Department of Pharmacology and Toxicology, University of Toronto, Toronto, ON, Canada; ^3^ InterVivo Solutions Inc., Mississauga, ON, Canada; ^4^ Leslie Dan Faculty of Pharmacy, University of Toronto, Toronto, ON, Canada; ^5^ DL Global Partners Inc., Toronto, ON, Canada

**Keywords:** psilocybin, motivation, depression, ketamine, microdose, attention, plasma exposure, psychedelic

## Abstract

Long term benefits following short-term administration of high psychedelic doses of serotonergic and dissociative hallucinogens, typified by psilocybin and ketamine respectively, support their potential as treatments for psychiatric conditions such as major depressive disorder. The high psychedelic doses induce perceptual experiences which are associated with therapeutic benefit. There have also been anecdotal reports of these drugs being used at what are colloquially referred to as “micro” doses to improve mood and cognitive function, although currently there are recognized limitations to their clinical and preclinical investigation. In the present studies we have defined a low dose and plasma exposure range in rats for both ketamine (0.3–3 mg/kg [10–73 ng/ml]) and psilocybin/psilocin (0.05–0.1 mg/kg [7–12 ng/ml]), based on studies which identified these as sub-threshold for the induction of behavioral stereotypies. Tests of efficacy were focused on depression-related endophenotypes of anhedonia, amotivation and cognitive dysfunction using low performing male Long Evans rats trained in two food motivated tasks: a progressive ratio (PR) and serial 5-choice (5-CSRT) task. Both acute doses of ketamine (1–3 mg/kg IP) and psilocybin (0.05–0.1 mg/kg SC) pretreatment increased break point for food (PR task), and improved attentional accuracy and a measure of impulsive action (5-CSRT task). In each case, effect size was modest and largely restricted to test subjects characterized as “low performing”. Furthermore, both drugs showed a similar pattern of effect across both tests. The present studies provide a framework for the future study of ketamine and psilocybin at low doses and plasma exposures, and help to establish the use of these lower concentrations of serotonergic and dissociative hallucinogens both as a valid scientific construct, and as having a therapeutic utility.

## Introduction

Major depressive disorder (MDD) is a significant global mental health condition. In the US alone, in 2017 an estimated 11 million adults had at least one major depressive episode with a severe impairment, an incidence that represented 4.5% of all US adults (https://www.nimh.nih.gov/health/statistics/major-depression.shtml). MDD is often a chronic, recurrent condition that adversely affects the quality of life of both the sufferer and their family and is associated with high levels of functional disability. According to DSM-V criteria, a diagnosis of MDD requires the individual to be experiencing five or more symptoms during the same 2-week period, with at least one symptom of either depressed mood or loss of interest or pleasure (anhedonia). Other criteria include fatigue/loss of energy (anergia), a slowing of thought and reduction of physical movement, a diminished ability to think or concentrate, or indecisiveness, significant weight loss unrelated to dieting, and feelings of guilt, worthlessness and suicidal ideation (van Loo et al., 2012; American Psychiatric Association, 2013).

The primary form of treatment for MDD is medication treatment combined with support by a healthcare professional. Second-generation antidepressants (e.g., selective serotonin reuptake inhibitors [SSRI’s] or selective serotonin norepinephrine reuptake inhibitors [SNRI’s]) represent the most widely used treatments, and most evidence-based guidelines recommend these medications as a first-line therapy (Kennedy et al., 2016; Gartlehner et al., 2017; Cipriani et al., 2018). Despite this recommendation, there are limitations to these treatments. For example, SSRI’s are associated with side effects such as sexual dysfunction, weight gain, and are increasingly recognized to poorly treat certain symptoms, such as anhedonia and amotivation (Fava and Kendler, 2000; Nutt et al., 2007; Furukawa et al., 2019). A substantial proportion of patients also fail to respond to SSRI therapy (28–55%) and the onset of antidepressant efficacy is typically delayed by weeks (Frazer and Benmansour, 2002).

The NMDA antagonist ketamine (Lodge and Mercier, 2015) has been demonstrated to have a rapid onset antidepressant property (Mathews et al., 2012; Corriger and Pickering, 2019), an observation that lead to FDA approval of an intranasal form of its S-(+) enantiomer (esketamine) for treatment resistant MDD in 1Q2019 (https://www.fda.gov/news-events/press-announcements/fda-approves-new-nasal-spray-medication-treatment-resistant-depression-available-only-certified). Esketamine, in conjunction with an oral antidepressant, has been shown to be effective in patients with treatment resistant MDD, with a rapid onset (i.e. within 24–48 h, Popova et al., 2019), although it is recognized that the optimum dose, duration and treatment frequency are not fully understood (Salahudeen et al., 2020). A modest incidence of side effects including dizziness, sedation, nausea, attentional disturbance, hypertension is reported at the approved doses (Morrison et al., 2018; Popova et al., 2019; Salahudeen et al., 2020). Because of the high relative incidence of dissociation and sedation associated with intranasal esketamine, the FDA has issued a boxed warning, and recommends that patients should be monitored for at least 2 h after drug administration. The medication is only available through a restricted Risk Evaluation and Mitigation Strategy (REMS) program due to concerns around sedation, and potential misuse and abuse for its dissociative and hallucinogenic effects (Salahudeen et al., 2020).

Over the last decade, the psychedelic drug class, notably psilocybin, has also been investigated as a treatment approach to MDD (Nichols, 2016; Geiger et al., 2018; Nutt, 2019). At psychotomimetic doses, psilocybin has been reported to produce a rapid relief of depressive symptoms in patients with treatment resistant, moderate to severe depression (Carhart-Harris et al., 2016) and also in patients with life-threatening cancer (Griffiths et al., 2016; Ross et al., 2016). These clinical studies have utilized two to three treatment sessions plus intensive personal support and therapist counseling and demonstrated antidepressant efficacy that persists beyond the pharmacodynamic/pharmacokinetic property of psilocybin, suggesting a long-term neuroadaptation or a reset of the “default networks” (Carhart-Harris et al., 2012; Kyzar et al., 2017; Nutt, 2019; Vollenweider and Preller, 2020). As a result of these outcomes, both the European (EMA) and US (FDA) regulatory bodies have approved larger, multicenter studies of psilocybin in MDD and other mental disorders which are ongoing and should report in 2021 (Nutt, 2019). Despite their differing pharmacological properties, both ketamine-based and psychedelic drug class treatments are being considered as important advances in the treatment of MDD.

In addition to usage at psychotomimetic doses, both ketamine and psilocybin have a long history of being self-adminstered at lower doses (colloquially referred to as “microdoses”), (Johnstad, 2018; Kuypers et al., 2019; Hutten et al., 2019; Polito and Stevenson, 2019; Rosenbaum et al., 2020). However, while there are a number of anecdotal reports describing beneficial effects of “micro” dose ketamine and psilocybin on mood and cognition, there are very few placebo controlled, scientific studies explicitly designed to investigate low doses of ketamine, and none explicitly using psilocybin (Kuypers et al., 2019; Kuypers, 2020). In the case of psilocybin, the actual doses are unknown since natural mushroom containing the agent are ingested. Furthermore, there is no scientific consensus as to the definition of “microdosing” (Kuypers et al., 2019). With self-administration of substances that are illegal in most jurisdictions, both the substance purity and its precise dose are impossible to determine, and without controlled trials, effects could be due to expectation and placebo response (Kuypers et al., 2019). Further, preclinical studies (Horsley et al., 2018; Meinhardt et al., 2020) have failed to show benefit of psilocybin and ketamine at doses designed to correspond to a human “microdose” experience.

The review article of Kuypers et al., (2019) highlighted some other key knowledge limitations around the microdosing of psychedelics, and by extension ketamine. One area that is lacking is the preclinical pharmacologic investigation of low dose psilocybin and ketamine, with a view to identifying behavioral changes that may translate to clinical efficacy, and understanding the mechanisms that may underlie these effects. A further question relates to the safety aspects of microdosing. In contrast to psychedelic doses of ketamine and psilocybin which may only be administered on a restricted schedule, “microdoses” are likely to be taken on a significantly more frequent basis and thus have a different safety profile.

It was a purpose of the present series of experiments to define dose and plasma concentration levels devoid of psychomimetic effects and then investigate the effects of these low doses of ketamine and psilocybin on two behavioral domains relevant to depression, namely 1) attention and response control as measured in the 5-choice serial reaction time task (Robbins, 2002; Higgins and Silenieks, 2017), and 2) motivation as measured using a progressive ratio schedule of food reinforcement (Hodos, 1960; Der-Avakian et al., 2016). Both tests map to specific endophenotypes associated with depression and which are translatable between the preclinical and clinical setting. Initial studies were focused on ketamine which was in turn guided by previous experience with the non-competitive NMDA antagonist dizocilpine (MK801; Wong et al., 1986). We, and others, have previously reported on positive pro-cognitive effects of low doses of dizocilpine measured in rats trained to perform the 5-choice task (Benn and Robinson, 2014; Guidi et al., 2015; Higgins et al., 2016), and we applied a similar approach to study ketamine and then psilocybin. In the present studies, a “low” dose range for each drug was first defined by identifying doses that were sub-threshold to those that induced behavioral stereotypies (i.e. head weaving/circling in ketamine; wet dog shakes in psilocybin) which have been described as a proxy for the psychomimetic effects of each drug class (Willetts et al., 1990; Hanks and Gonzalez-Maeso, 2013; Halberstadt and Geyer, 2018). To counter the potential for any marginal effects of the low doses tested, we adopted a relatively large study sample size and subgrouping of rats based on performance in an attempt to improve test sensitivity (Button et al., 2013; Hayward et al., 2016). Furthermore, we evaluated the plasma levels of ketamine and psilocybin at doses relevant to the present studies.

## Methods

### Animals and Housing

Male Long Evans rats were used in all experiments (source: Charles River Laboratories, St. Constant, QE, Canada). All study animals were singly housed in a vivarium under a 12 h light:dark cycle (lights on: 06:00 h–18:00 h). All behavioral testing was during the light phase. Rats utilized for the overt behavioral experiments had ad-lib access to food and water. Rats utilized for the 5-choice and progressive ratio experiments were placed on a restricted diet regimen throughout training and testing, where they were fed once a day with approximately 18–20 g of standard laboratory chow at the completion of testing procedures (16:00 h–18:00 h). All animal use procedures were performed in accordance with the principles of the Canadian Council on Animal Care (CCAC).

### Drugs and Treatment Regimens

Psilocybin (lot #: AAT-06-61-010419; purity 97%; source Dalton Pharma Services, Toronto, ON, Canada) was suspended in 0.9% saline and sonicated until fully dissolved. Drug was administered in a volume of 1 ml/kg, subcutaneous (SC) route. (±) Ketamine hydrochloride (Vetoquinol; 100 mg/ml) was suspended in 0.9% saline and administered at a volume of 1 ml/kg, via the intraperitoneal (IP) route. M100907 HCl (volinanserin; Toronto Research Chemicals, North York, Ontario, Canada) was dissolved in 0.9% saline solution containing 0.3% Tween and injected SC, 30 min pre-psilocybin or vehicle. Dizocilpine maleate (MK801; source: Tocris Chemicals, Bristol, United Kingdom), was administered SC in a volume of 1 ml/kg. Doses are expressed as that of base. Pretreatment times for psilocybin, ketamine and dizocilpine was 10 min, except in the observation test when test onset was immediately post injection. A 10 min pretreatment time was adopted for the PR and 5-choice tests to ensure significant drug exposure at test onset.

### Effect of Ketamine and Psilocybin on Overt Spontaneous Measures of Behavior

A total of 30 male Long Evans rats were used for these experiments (N = 15 Ketamine study; N = 15 psilocybin study). Rats were dosed in a semi-random sequence with test drug or vehicle control every 4–5 days. Immediately post-dose, the subjects were placed in automated activity test chambers (17″ W x 17″ L × 12″ H; Med Associates) for 2 h. During the session, spontaneous activity including distance traveled, rearing activity, and ambulatory episodes were recorded. For the duration of the 2 h session, animals were also monitored by trained technicians for behaviors specific to the drug being tested.

Ketamine: Ketamine doses 0.3, 1, 3, 10, 30, 60 mg/kg, or dizocilpine (MK801; 0.1 mg/kg SC), or vehicle were investigated. In addition to locomotor activity measures, animals were monitored by trained technicians for the following behaviors consistent with NMDA channel block: head weaving, circling, body sway, ataxia (Tricklebank et al., 1989). Behaviors were scored every 5 min as 0 = absent, or 1 = present during a 10 s visual assessment, i. e a maximum score of 24.

Psilocybin: Psilocybin doses 0.03, 0.1, 0.3, 1, 3, 10 mg/kg, or vehicle (saline) SC were investigated. In addition to locomotor activity measures, animals were continuously monitored by trained technicians for behavioral parameters characteristic of 5-HT2A receptor activation: (wet dog shakes (WDS), back muscle contractions (BMC)), 5-HT2C receptor activation (yawning, penile grooming (YPG)), and 5-HT1A receptor activation (Forepaw treading (FPT), hindlimb abduction (HLA)) (see Haberzettl et al., 2013 for review) for the duration of the 2 h session. At the completion of the dose-response study, an additional test was conducted in which animals received a single administration of the 5-HT2A receptor antagonist M100907 (0.5 mg/kg, IP) or vehicle control 30 min prior to administration of psilocybin (1 mg/kg) in a cross-over design. Subjects were placed in the activity chambers and measures taken as described above.

### Pharmacokinetic Analysis of Ketamine and Psilocybin/Psilocin

Doses of ketamine and psilocybin selected for PK evaluation were determined based on outcomes from the overt behavioral experiments and selected to include 1) dose(s) sub-threshold for eliciting behavioral signs, and 2) dose(s) that reliably induced such signs. A total of 35 male Long Evans rats were used for this study.

Fifteen rats were randomly allocated to three groups (N = 5/group) and treated with ketamine at doses of 0.3, three or 30 mg/kg IP. At 0.25, 0.5, 0.75, 1, 1.5, 2, 3 h post treatment, ∼100 μL whole blood was collected via saphenous vein bleed into K2EDTA capillary tubes.

Twenty rats were allocated to four groups (N = 5/group) and treated with psilocybin at doses of 0.05, 0.1, one or 10 mg/kg SC. At 0.25, 0.5, 0.75, 1, 2, 4, and 6 h post-treatment, ∼100 μL whole blood was collected via saphenous vein bleed into K2EDTA capillary tubes.

All blood samples were kept on wet ice for a maximum of 5 min prior to centrifugation (3,300 rpm at 4°C, 5 min). Plasma was extracted and immediately frozen at −20°C before transfer to −80°C, where stored until shipment to the InterVivo Bioanalytical facility. for the determination of plasma concentrations of ketamine (ketamine study) or psilocybin and its primary metabolite psilocin (psilocybin study).

### Quantification of Ketamine, Psilocybin, and Psilocin by LC-MS/MS

Plasma samples were analyzed by an AB Sciex API4000 QTRAP liquid chromatography/mass spectrometric system equipped with an ESI source in positive ion mode and coupled to an Agilent 1,200 liquid chromatographic system. Ketamine hydrochloride, ketamine-d4 hydrochloride, psilocin, psilocybin, psilocin-d10 and procainamide were purchased from Sigma-Aldrich.

For ketamine quantification, 50/50 methanol/acetonitrile containing internal standard (IS), ketamine-d_4_ was added to plasma sample (5 µL) to precipitate proteins. The supernatant was diluted with mobile phase for injection prior to analysis. Chromatographic separation was performed on a Zorbax XDB-C18 column (2.1 × 30 mm, 3.5 µm) at 25°C using gradient elution (mobile phase: 10 mM ammonium formate, pH 3.0 (A) and 95% methanol/5% 10 mM ammonium formate, pH 3.0 (B)). The multiple reaction monitoring (MRM) parameters for ketamine and IS were m/z 238.1 to 125.1 (quantitative)/220.2 (qualitative) and m/z 242.1 to 129.1, respectively. The calibration dynamic range was 0.5–5,000 ng/ml.

For psilocybin and psilocin quantification, 50/50 methanol/acetonitrile containing IS (procainamide and psilocin-d10 for psilocybin and psilocin, respectively) was added to plasma (20 µL). The supernatant was diluted with 0.1% formic acid in water prior to analysis. Two serially connected Javelin Aquasil C18 columns (2.1 × 20 mm, 5 µm each) at 25°C were used with gradient elution (mobile phase: 0.1% formic acid in water (A) and 0.1% formic acid in methanol (B)). The MRM parameters for psilocybin, psilocin, psilocin-d10 and procainamide were m/z 285.09 to 58.2, m/z 205.2 to 58.2, m/z 215.2 to 66.2 and m/z 236.1 to 163.0, respectively. The calibration range was 0.5–200 ng/ml for psilocybin and 0.1–200 ng/ml for psilocin.

### Effect of Ketamine and Psilocybin on Motivation for Food: Progressive Ratio Task

Operant test chambers (Med Associates Inc., St. Albans, VT) were housed in sound-insulated and ventilated enclosures. Each chamber consisted of an aluminum enclosure (25 × 30 cm), containing on one wall a food hopper and house light, with a response lever and a stimulus light positioned each side of the hopper. Chambers were controlled by Med PC software using programs written in-house (Med Associates Inc., St. Albans, VT).

A total of 72 rats were trained to lever press for a food reward (45 mg Bioserv pellets) under a progressive ratio (PR) schedule in which the number of responses required to achieve a pellet increases for successive reinforcers. The progressive ratio was derived from the equation ratio = [5 × e(0.2 × reinforcer #)–5], and was as follows: 2, 4, 6, 9, 12, 15, 20, 25, 32, 40, 50, 62, 77, 95, 118, 145, etc. The break point, which is the primary measure for this task, was determined when the rat failed to earn a pellet in 20 min, providing a quantitative measurement for motivation. Once the rats reached a stable level of responding, defined as when individual break points did not vary by >15% over three consecutive sessions, the effect of ketamine (0.3–6 mg/kg IP; N = 68 rats) and psilocybin (0.025–0.1 mg/kg SC; N = 72 rats) was examined. The group size difference was due to four rats being redeployed during the study. In approximately half of the rats, ketamine was tested first, followed by psilocybin, and the other half in reverse order. Subjects were administered drug treatments or vehicle control according to Latin square design, in which each animal received each treatment over repeated test sessions with 3–4 days intervals between each testing cycle. Recorded measures included break point (number of pellets earned), total lever presses made during the test session, and test session duration.

Subjects were also ranked based on pellets earned and total lever presses over a 7-days pretest period, and the lowest tertile were classified as low responders (N = 23 ketamine study; N = 24 psilocybin study). Treatment effects were analyzed both in the entire study cohort, and in the low responder subgroup. At the completion of drug testing, two assessments were made to compare free feeding (latency to consume 15 × 45 mg food pellets, and the number of 45 mg pellets eaten in a 3 min free feeding session) and locomotor activity (60 min open field activity test) between the high and low tertile groups. Body weights were also compared.

### Effect of Ketamine and Psilocybin on Attention and Response Control: 5-Choice Task

Test system was twelve 5-choice operant chambers (Med Associates Inc., St. Albans, VT). Each consisted of an aluminum enclosure (25 × 30 cm), containing on one wall a reward magazine attached to a food pellet dispenser, and house light, and on the opposite wall an array of five square niches (2.5 × 2.5 × 2.5 cm) arranged on a curved panel. An LED was positioned at the rear of each niche. Each niche, and the reward magazine, also contained a photocell to detect head entry. Test chambers were controlled by Med PC software (Med Associates Inc., St. Albans, VT).

Each 5-CSRTT session began with the illumination of the house light and delivery of a food pellet (45 mg food pellet, Bioserv, USA). A nose-poke into the magazine tray initiated the first trial which consisted of an inter-trial interval (ITI, 5 s) followed by the random illumination of one of the five lights for a fixed interval (stimulus duration, SD). If a nose-poke was registered in the illuminated niche before the end of either the SD, or a fixed interval after this period (limited hold, LH) a further pellet was dispensed and a Correct Trial registered. An incorrect nose poke (Incorrect Trial) or failure to respond within the allotted time (Missed Trial) resulted in a Time Out (TO) period in which the houselight was extinguished for 5 s. Responding into one of the five niches during the ITI (premature response; PREM) resulted in a further TO. Perseverative responses (PSV), which were responses made after a correct response but prior to food collection were also recorded but not punished by TO.

Each session ran for either 100 trials or 60 min, whichever endpoint was achieved first. Initially, stimulus parameters were such that SD was set at 60 s, and ITI, TO, and LH were 5 s. For all subjects the SD was progressively reduced until a final duration of 0.75 s was achieved. All other parameters remained at their initial levels throughout training and test, except during the extended ITI schedule (see below). Training continued under the target stimulus parameters until subjects had achieved consistent performance above a threshold of approximately 70% correct ([correct/(correct + incorrect)] * 100) and <20% omissions for at least a two-week period. At this point drug testing began according to a repeated measures design with animals tested twice weekly (either Tuesday/Friday or Wednesday/Saturday) and run under baseline conditions over intervening days. Two test conditions were used:

Standard test conditions (Study 1)–The effect of ketamine (0.3–6 mg/kg IP; N = 36 rats) and psilocybin (0.05–0.1 mg/kg SC; N = 24 rats) was evaluated under standard test conditions of 0.75 s SD, 5 s ITI, 5 s LH, 100 trials. Drug testing was conducted according to a Latin square design in which each animal received each dose of test drug or vehicle over repeated test sessions. A subgrouping was made in this experiment based on baseline % correct made over 5 days preceding these experiments. The effect of treatment in the upper (high performer) and lower (low performer) subgroups (N = 12/subgroup ketamine study; N = 8/subgroup psilocybin study) was investigated, in addition to the entire study cohort. Separate groups of rats were used for each drug.

Long 10 s ITI (Study 2)–The effect of ketamine (1–6 mg/kg IP; N = 24 rats) and psilocybin (0.025–0.1 mg/kg SC; N = 57 rats) was evaluated under the test challenge condition of reduced stimulus duration (0.3 s SD), extended ITI (10 s ITI). Limited hold (5 s) and trial number (100 trials) were same as for standard condition. Drug testing was conducted according to a Latin square design in which each animal received each dose of test drug or vehicle over repeated test sessions. A subgrouping was made in this experiment based on the number of PREM responses made following vehicle pretreatment under the 10 s ITI condition. The effect of treatment in the upper (high impulsive, HI) and lower (low impulsive, LI) subgroups was investigated (Ketamine study: N = 8 per tertile; Psilocybin study: N = 19 per tertile), in addition to the entire study cohort.

### Statistical Analysis

Data from the 5-CSRTT and progressive ratio tasks was analyzed by one way (treatment) repeated measures ANOVA (Statistica Version 11, Statsoft Inc. [2012]). In the event of a significant main effect, post-hoc comparisons were carried out with Dunnett’s test for comparison of drug treatment to vehicle control.

Test subjects in the 5-choice experiments were divided into high and low performance groups based on (A) % correct measure following vehicle pretreatment (5-choice, standard conditions), or (B) number of PREM responses measured under the long 10 s ITI schedule following vehicle pretreatment (10 s ITI test conditions). Each low and high group consisted of the extreme tertile rats; the middle tertile group was excluded from this analysis. Attentional accuracy was measured both as % correct (#correct/#correct+#incorrect*100) and % hit (#correct/#correct+#incorrect+#omissions*100). In all cases the accepted level of significance was *p* < 0.05. Effect sizes for group mean differences were also calculated using partial eta squared (Statistica Version 11, Statsoft Inc. [2012]).

The 5-choice studies were conducted on a single occasion, i.e. classical block design. However, the progressive ratio experiments were run as two separate block experiments and the data pooled to increase the overall sample size based on a standardized test design and dosing schedule. Such an adaptive sequential study design has been proposed as an approach to improve experimental efficiency and to counter studies which are underpowered (Button et al., 2013; Neumann et al., 2017).

Plasma concentrations were analyzed, where appropriate, by noncompartmental methods using Phoenix® WinNonlin® 8.2 (Pharsight, Certara, Mountanview, CA). PK parameters and plasma concentrations are reported as the mean ± S.D.

## Results

### Effect of Ketamine and Psilocybin on Overt Spontaneous Measures of Behavior

#### Ketamine

**FIGURE 1:**
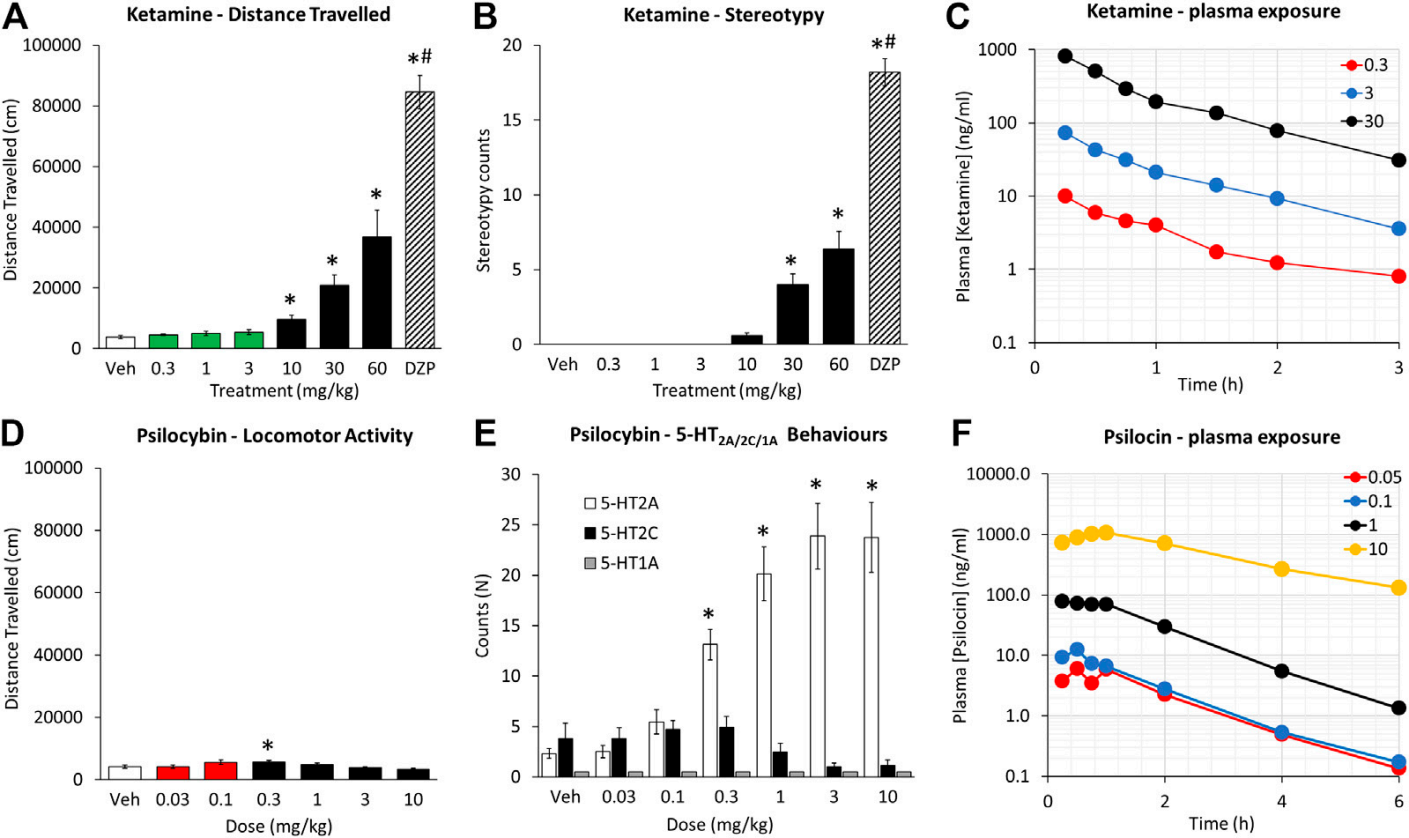
Characterisation of ketamine (0.3–60 mg/kg IP) and psilocybin (0.03–10 mg/kg SC) on locomotor activity, measured as distance traveled over a 2 h test period **(A and D)**, behavioral stereotypies recorded over the same test period **(B and E)**, and plasma [ketamine] and [psilocin] levels as a function of time post injection of ketamine (0.3–30 mg/kg IP) and psilocybin (0.05–10 mg/kg SC) **(C and F)**. Data for distance traveled is presented on the same axis scales to emphasize the differences in magnitude of effect between the two drugs. The green and red highlighted bars are to emphasize the dose ranges of ketamine and psilocybin that were defined as low doses, and used for the progressive ratio and 5-choice experiments. Behavioral signs characterized as 5-HT2AR were a composite of WDS/BMC, 5-HT2CR a composite of YPG, 5-HT1AR a composite of HLA/FPT (see Halberzettl et al., 2013). Dizocilpine (DZP; 0.1 mg/kg SC) was included as a reference comparator in the ketamine experiment. *p < 0.05 vs. vehicle control, #*p* < 0.05 vs. all treatment groups (see Data analysis section).

Ketamine produced a dose related increase in the incidence of overt behaviors and locomotion with a threshold dose of 10 mg/kg (see Figures 1A,B). The principal behavioral components recorded were circling and ataxia, although occasional measures of body sway and head weaving were noted. The overall incidence of these signs was scored significantly lower than dizocilpine (0.1 mg/kg), run as a reference comparison. This was due to dizocilpine (0.1 mg/kg) having both a longer duration of action and inducing these signs with greater intensity. While dizocilpine effects on behavior were still evident at 2 h post treatment, the effects of the highest ketamine dose (60 mg/kg) had largely subsided by 90 min.

#### Psilocybin

Psilocybin (0.03–10 mg/kg) produced a dose related increase in the incidence of behaviors presumed to be 5-HT2A receptor mediated (i.e. WDS/BMC) with a threshold dose of 0.3 mg/kg (see Figure 1E). While BMC showed a monotonic increase with dose (1–10 mg/kg), WDS showed a biphasic dose response peaking at 1 mg/kg, and declining slightly at 3–10 mg/kg. There was no main treatment effect of psilocybin on behaviors related to 5-HT2C (YPG) or 5-HT1A (i.e. FPT/HLA) receptor activation. Analysis of locomotor activity revealed main effects of treatment on distance traveled (F6,84 = 4.4; *p* < 0.01) and rearing counts (F6,84 = 6.2, *p* < 0.01), which reflected an modest increase in distance traveled at the 0.1–0.3 mg/kg dose range, and decrease in rearing counts at the 10 mg/kg dose relative to vehicle pretreatment (see Figure 1D). It should be noted that the increase in distance traveled following psilocybin 0.1–0.3 mg/kg pretreatment (Veh: 4,141 ± 497; psilo 0.1 mg/kg: 5,560 ± 770; *p* = 0.08; psilo 0.3 mg/kg: 5,716 ± 399; *p* = 0.04) was significantly less that induced by ketamine (10–30 mg/kg) and was not accompanied by behavioral stereotypy.

In a follow-up study, the induction of WDS/BMC induced by psilocybin (1 mg/kg) was completely blocked by pretreatment with the 5-HT2A receptor antagonist M100907 (0.5 mg/kg; Kehne et al., 1996) (Veh/Psilocybin: 35.9 ± 4.9; M100907/Psilocybin: 0.7 ± 0.3; *p* < 0.001). There was no change in the incidence of behaviors attributed to 5-HT2C receptor activation (Veh/Psilocybin: 2.3 ± 0.5; M100907/Psilocybin: 3.6 ± 1.2; NS). These results are consistent with a 5-HT2A receptor agonist property of psilocybin.

### Effect of Ketamine and Psilocybin on Motivation for Food: Progressive Ratio Task

The progressive ratio studies were conducted in a large cohort of rats (68–72) which enabled the subgrouping of rats into tertiles of “low” and “high” performers, based on their breakpoint/lever press measures recorded over 5 days baseline prior to testing. Particular interest was placed on the “low performer” subgroup on studying the effect of ketamine and psilocybin. In a series of control experiments conducted on the “low” and “high” tertile groups used in the psilocybin study, the groups were evaluated for locomotor activity in an open field, measures of free feeding (time to consume 15 pellets, which corresponded to maximum food intake in the PR test), and body weight. These data are summarized in Table 1 and indicate that despite having differences in food motivation based on availability under a PR schedule, the subgroups have similar open field activity, a measure of free feeding and body weights.

**TABLE 1:** Comparison between “high” and “low” PR responders (N = 23 rats per tertile; N = 72 rats total, i.e. “All”) on a measure of free feeding, open field activity and body weight. Measures of free feeding was the latency to consume 15 × 45 mg food pellets, and the number of 45 mg pellets eaten in a 3 min free feeding session (maximum number = 15 which approximated to the amount of food pellets earned in a progressive ratio test session. Open field activity was measured as the distance traveled and the number of rears counted in a 60 min test. Body weights were also compared between the start and completion of testing. There were no differences between “high” and “low” PR responders on any of these measures, despite robust differences in no. lever presses and break point. *p < 0.01 vs. “High” responders.

Subgroup	Latency to consume 15 pellets	Pellets eaten in 3 min	Distance travelled	Rearing counts	Body weight: Start	Body weight: End	Lever press	Break point
All (N=72)	52.1 ± 3.4	15	7058 ± 375	109 ± 5	423 ± 4	423 ± 5	345 ± 22	11.5 ± 0.3
“High”reponders (N=23)	46.5 ± 4.1	15	7503 ± 732	102 ± 11	425 ± 5	426 ± 5	561 ± 24	13.9 ± 0.1
“Low”reponders (N=23)	49.7 ± 5.1	15	6725 ± 609	94 ± 13	431 ± 4	432 ± 3	173 ± 14*	9.2 ± 0.3*

#### Ketamine

**FIGURE 2:**
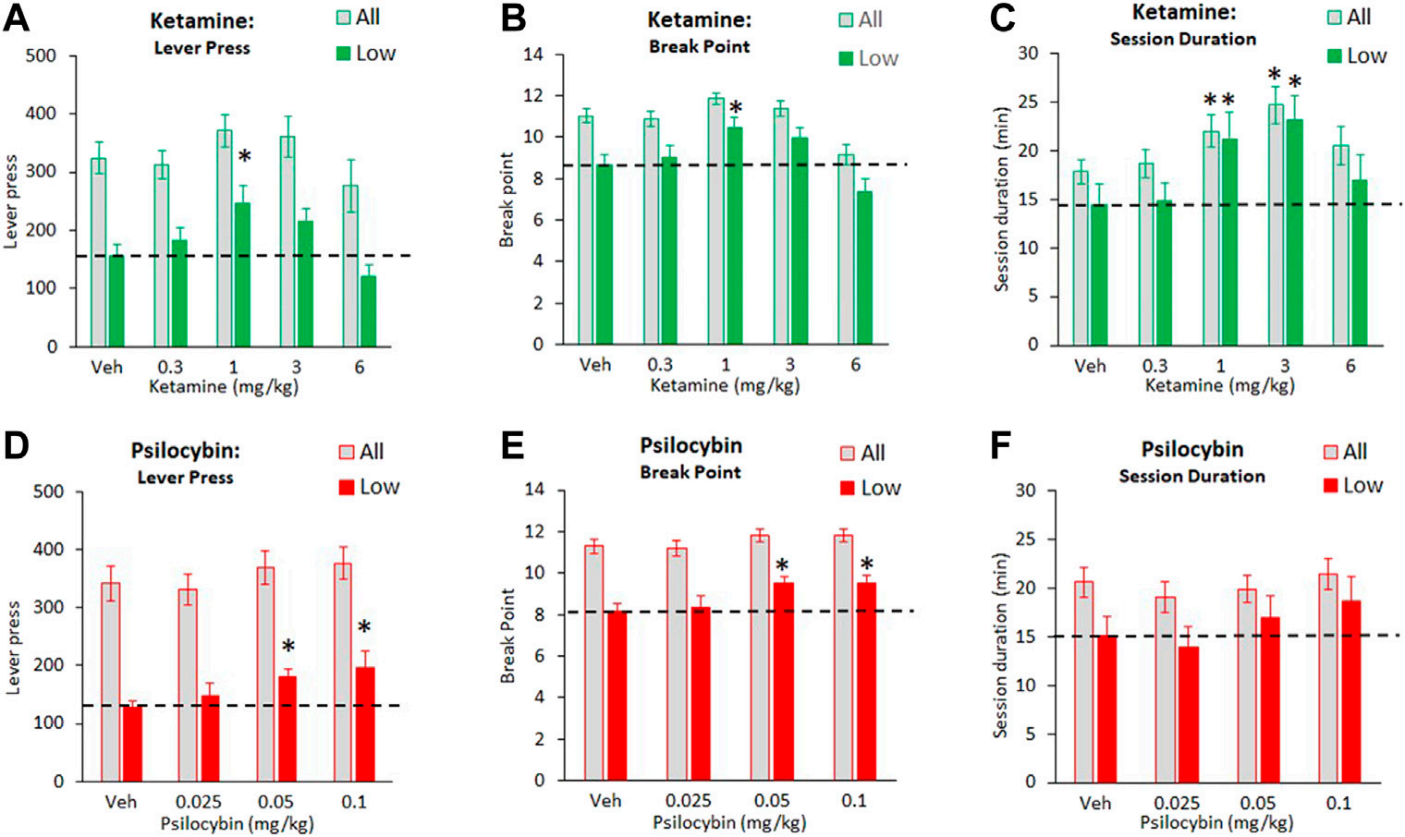
Characterisation of ketamine (0.3–6 mg/kg IP) and psilocybin (0.025–0.1 mg/kg SC) on food responding made available under a progressive schedule of reinforcement. Data is presented for both drugs as total number of lever presses recorded during the test session **(A and D)**, final break point **(B and E)**, and total session duration **(C and F)**. Data for each drug is presented both as all test subjects (ketamine: N = 68; psilocybin: N = 72), and subjects characterized as “low performers” based on having the lowest tertile on lever presses/break point based on performance measured over 7 days prior to onset of drug testing (ketamine: N = 23; psilocybin: N = 24). The hashed line is to highlight the level of the “low performer” subgroup following vehicle pretreatment. *p < 0.05 vs. vehicle control (Dunnetts test following significant ANOVA).

The effect of ketamine (0.3–6 mg/kg) on PR responding for food was assessed in a cohort size of 68 rats total. Despite main effects of treatment on number of lever press (F4,268 = 2.9, *p* = 0.02; ƞ_p_^2^ = 0.04), no treatment group was significantly different to vehicle. Main effect of treatment on break point (F4,268 = 13.3, *p* < 0.01; ƞ_p_^2^ = 0.16) reflected a decrease in this measure at the 6 mg/kg dose, and a main effect of treatment on session duration (F4,268 = 5.2, *p* < 0.01; ƞ_p_^2^ = 0.07) reflected an increase in this measure following ketamine 1–3 mg/kg doses (see Figures 2A–C).

A clearer pattern of ketamine effect was noted in the “low responder” subgroup (N = 23 rats) where main effects of treatment on lever press (F4,88 = 6.6, *p* < 0.01; ƞ_p_^2^ = 0.23), break point (F4,88 = 8.9, *p* < 0.01; ƞ_p_^2^ = 0.29) and session duration (F4,88 = 5.0, *p* = 0.01; ƞ_p_^2^ = 0.19) reflected a clear trend, and in most cases statistically significant effect, of ketamine 1–3 mg/kg doses to increase each measure relative to vehicle pretreatment (see Figures 2A–C). At the 6 mg/kg dose, there was a trend for a return to vehicle baseline levels of performance, i. e an inverted “U” shaped dose response.

#### Psilocybin

The effect of psilocybin (0.025–0.1 mg/kg) on PR responding for food was determined in a cohort size of 72 rats total. Assessed over all 72 rats, there was no main effect of treatment on number of lever press (F3,210 = 1.9, *p* = 0.1; NS; ƞ_p_^2^ = 0.03), or session duration (F3,210 = 1.2, NS; ƞ_p_^2^ = 0.02). A main effect of treatment was recorded for break point (F3,210 = 3.3; *p* = 0.02; ƞ_p_^2^ = 0.05), however no treatment group was significantly different to vehicle (see Figures 2D–F).

Analysis confined to the “low responder” cohort (N = 24 rats) revealed main effects of treatment on lever press (F3,69 = 4.9, *p* < 0.01; ƞ_p_^2^ = 0.17) and break point (F3,69 = 5.6; *p* < 0.01; ƞ_p_^2^ = 0.19), reflecting increases in both measures following psilocybin 0.05–0.1 mg/kg pretreatment relative to vehicle. There was no treatment effect on session duration (F3,69 = 2.4; *p* = 0.07, NS; ƞ_p_^2^ = 0.09), although there was trend for this to be increased at the 0.1 mg/kg dose (Veh: 15.1 ± 2.0 min; Psilo 0.1 mg/kg: 18.7 ± 2.5 min) (see Figures 2D–F).

### Effect of Ketamine and Psilocybin on Attention and Response Control: 5-Choice Task

#### Ketamine

##### Standard Conditions (5 s ITI, 0.75 s SD)

Ketamine was tested at 0.3–6 mg/kg in a total of 36 rats. Main effects of treatment on % hit, omissions, premature responses (all F4,140 > 5.1, *p* < 0.01; ƞ_p_^2^>0.13) reflected effects at the 6 mg/kg ketamine dose to reduce % hit, and to increase omissions, premature and perseverative responses relative to vehicle pretreatment. Lower doses had no significant effects on these measures. There was no main effect on % correct or total trials (F4,140 < 1.5, NS; ƞ_p_^2^<0.04) (see Table 2 for summary).

**TABLE 2:** Performance measures from 5-choice serial reaction time task: Standard conditions of 0.75 s SD, 5 s ITI, 100 trials. Data is presented for all rats treated with ketamine (N = 36) or Psilocybin (N = 24). *p < 0.05 vs. vehicle.

Treatment	#Correct trails	#Incorrect trails	#Missed trails	% Correct	% Hit	PREM	PSV	Correct latency (s)	Magazine latency (s)	Total trails
Vehicle	69.5 ± 2.8	12.7 ± 1.3	6.1 ± 1.2	84.2 ± 1.7	78.6 ± 2.1	9.0 ± 1.4	25.1 ± 2.0	0.41 ± 0.01	1.38 ± 0.09	88.3 ± 2.4
Ketamine (0.3 mg/kg)	71.3 ± 2.1	13.9 ± 1.3	6.2 ± 1.1	83.3 ± 1.6	77.9 ± 1.2	7.4 ± 1.0	29.0 ± 2.5	0.42 ± 0.01	1.50 ± 0.11	86.9 ± 3.7
Ketamine (1 mg/kg)	70.6 ± 2.7	11.7 ± 1.1	7.0 ± 1.3	85.5 ± 1.5	78.7 ± 2.0	8.4 ± 1.5	25.4 ± 2.4	0.42 ± 0.01	1.33 ± 0.07	83.9 ± 4.1
Ketamine (3 mg/kg)	68.0 ± 3.1	11.1 ± 1.4	8.6 ± 1.5	85.4 ± 1.9	76.7 ± 2.5	9.2 ± 1.7	28.9 ± 2.6	0.40 ± 0.01	1.26 ± 0.08	87.7 ± 2.1
Ketamine (6 mg/kg)	53.8 ± 3.6*	10.0 ± 1.0	18.5 ± 2.4*	83.0 ± 1.8	65.5 ± 3.1*	14.0 ± 2.3*	40.3 ± 4.5*	0.34 ± 0.01*	1.05 ± 0.12	82.3 ± 2.8
Vehicle	67.8 ± 3.8	11.1 ± 1.1	15.41 ± 2.5	85.2 ± 1.5	71.3 ± 3.1	5.1 ± 0.8	20.8 ± 2.3	0.73 ± 0.03	3.04 ± 0.38	94.1 ± 2.3
Psilocybin (0.05 mg/kg)	72.1 ± 3.6	9.6 ± 1.2	14.5 ± 2.4	87.5 ± 1.6	74.5 ± 2.8	5.2 ± 0.9	21.3 ± 3.1	0.69 ± 0.03	3.42 ± 0.66	96.1 ± 2.7
Psilocybin (0.1 mg/kg)	70.0 ± 3.2	9.8 ± 1.2	15.8 ± 2.8	87.7 ± 1.5	73.4 ± 2.8	5.9 ± 1.1	25.5 ± 3.7	0.70 ± 0.03	3.97 ± 0.94	95.7 ± 2.4

**FIGURE 3:**
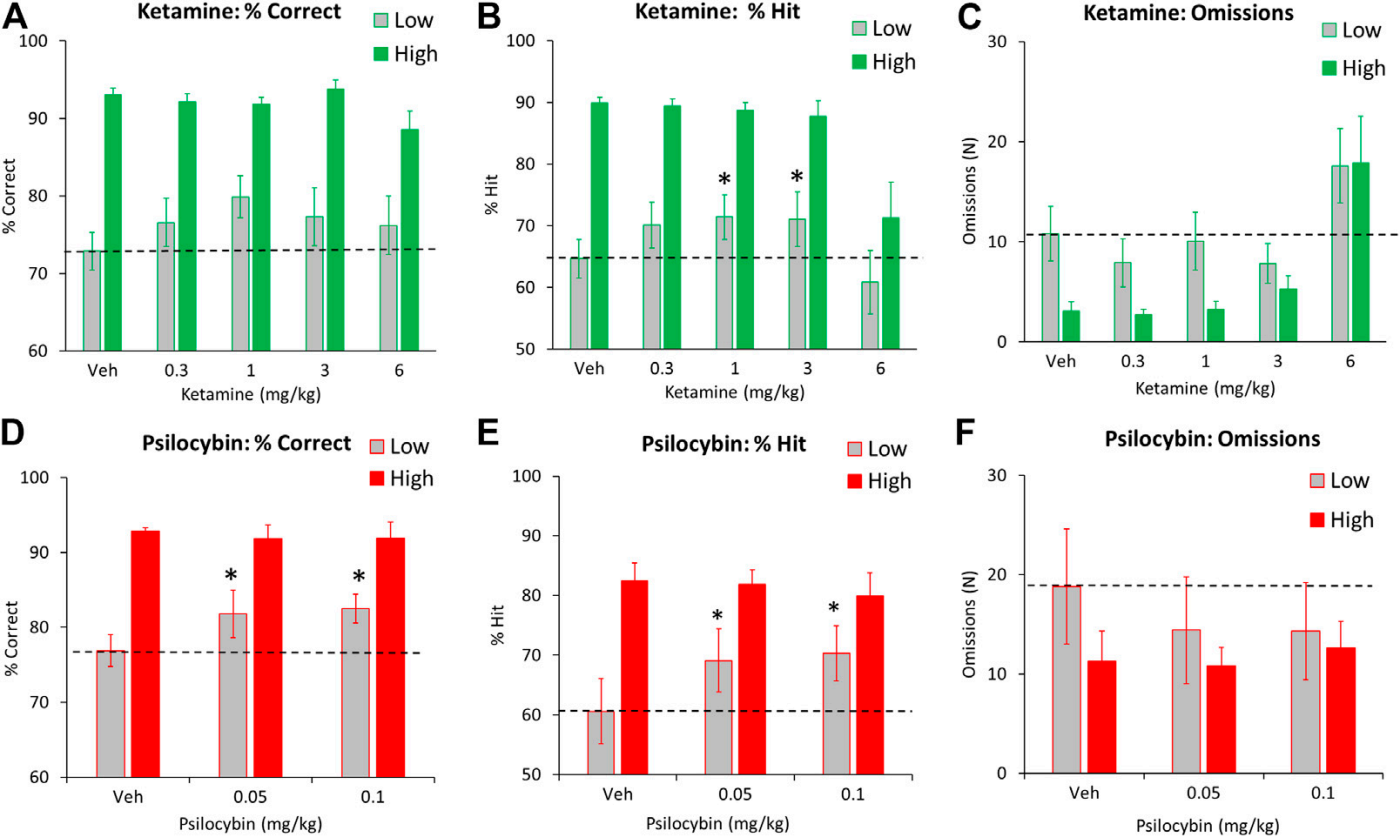
Characterisation of ketamine (0.3–6 mg/kg IP) and psilocybin (0.05–0.1 mg/kg SC) on performance of rats in the 5-choice serial reaction time task. Test conditions of 0.75 s SD, 5 s ITI, 100 trials. Data is presented for both drugs as % correct, **(A and D)**, % hit **(B and E)**, and omissions (missed/incomplete) trials **(C and F)**. Data for each drug is presented for subjects characterized as “high” and “low” performers (ketamine: N = 12 per tertile; psilocybin: N = 8 per tertile), based on extreme tertile groups ranked on % correct performance measured over 7 days prior to onset of drug testing. The hashed line is to highlight the level of the “low performer” subgroup following vehicle pretreatment. *p < 0.05 vs. vehicle control (Dunnetts test following significant ANOVA).

Subgrouping the rats into “low” and “high” performers based on % correct revealed main effects of treatment on omissions, premature and perseverative responses (all F4,88 > 4.6, *p* < 0.01; ƞ_p_^2^>0.17) but no treatment × subgroup interaction (F4,88 < 1.3, NS; ƞ_p_^2^<0.05) reflecting effects on these measures at the 6 mg/kg dose was recorded in both performance groups. A borderline interaction of treatment x subgroup was recorded on the accuracy measures of % correct (F4,88 = 2.3, *p* = 0.07; ƞ_p_^2^ = 0.09) and % hit (F4,88 = 2.1, *p* = 0.09; ƞ_p_^2^ = 0.09). A sub-analysis conducted on the low performing cohort only (N = 12) revealed main effects of treatment on % hit (F4,44 = 3.9, *p* < 0.01; ƞ_p_^2^ = 0.26), and correct latency (F4,44 = 3.9, *p* < 0.01, NS; ƞ_p_^2^ = 0.26) but not % correct (F4,44 = 1.8, NS; ƞ_p_^2^ = 0.14). This reflected a small improvement in accuracy at the 1 mg/kg and 3 mg/kg compared to vehicle pretreatment on % hit measure. Response speed was also faster in the 6 mg/kg ketamine group (Figures 3A–C and Table 3).

**TABLE 3:** Performance measures from 5-choice serial reaction time task: Standard conditions of 0.75 s SD, 5 s ITI, 100 trials. Ketamine “low” and “high” performers (N = 12 per subgroup).

Subgroup	Treatment	#Correct trials	#Incorrect trials	#Missed trials	%Correct	%Hit	PREM	PSV	Correct latency (s)	Magazine latency (s)	Total trials
Low performers	Vehicle	59.6 ± 3.4	21.6 ± 1.5	10.8 ± 2.7	72.9 ± 2.4	64.7 ± 3.1	8.0 ± 2.1	25.3 ± 2.8	0.39 ± 0.02	1.24 ± 0.13	92.0 ± 2.1
	Ketamine (0.3 mg/kg)	62.3 ± 3.6	18.9 ± 2.6	7.9 ± 2.4	76.6 ± 3.1	70.1 ± 3.7	7.4 ± 1.4	23.9 ± 2.7	0.41 ± 0.02	1.42 ± 0.18	89.2 ± 2.6
	Ketamine (1 mg/kg)	65.3 ± 3.9	16.0 ± 2.0*	10.1 ± 2.9	79.9 ± 2.7*	71.4 ± 3.6*	8.6 ± 2.5	25.6 ± 2.8	0.42 ± 0.02	1.18 ± 1.11	91.4 ± 2.5
	Ketamine (3 mg/kg)	63.6 ± 5.1	17.8 ± 2.8	7.8 ± 2.0	77.3 ± 3.7	71.0 ± 4.4	10.8 ± 3.3	31.5 ± 4.0	0.40 ± 0.03	1.16 ± 0.17	89.2 ± 3.3.
	Ketamine (6 mg/kg)	50.5 ± 5.2*	14.2 ± 2.0*	17.6 ± 3.7*	76.2 ± 3.8	60.9 ± 5.1	16.3 ± 4.1*	43.2 ± 8.0	0.34 ± 0.02*	0.98 ± 0.18*	82.3 ± 3.9*
High performers	Vehicle	81.2 ± 3.3	5.9 ± 0.7	3.1 ± 0.9	93.1 ± 0.9	89.9 ± 0.9	8.2 ± 3.2	24.6 ± 3.4	0.42 ± 0.02	1.68 ± 0.24	90.2 ± 3.2
	Ketamine (0.3 mg/kg)	83.1 ± 2.1	7.1 ± 1.0	2.8 ± 0.5	92.1 ± 1.0	89.4 ± 1.1	7.1 ± 2.1	31.8 ± 4.7	0.42 ± 0.02	1.70 ± 0.27	92.9 ± 2.1
	Ketamine (1 mg/kg)	82.7 ± 2.4	7.3 ± 0.8	3.3 ± 0.8	91.8 ± 0.9	88.6 ± 1.4	5.7 ± 1.8	26.3 ± 4.5	0.42 ± 0.02	1.55 ± 0.13	93.2 ± 1.9
	Ketamine (3 mg/kg)	78.2 ± 4.9	4.8 ± 0.8	5.3 ± 1.4	93.8 ± 1.2	87.8 ± 2.5	8.5 ± 3.3	26.6 ± 4.2	0.41 ± 0.02	1.36 ± 0.11	88.2 ± 4.1
	Ketamine (6 mg/kg)	62.0 ± 6.7*	6.5 ± 1.0	17.8 ± 4.7*	88.6 ± 2.3	71.3 ± 5.7	13.2 ± 5.2*	43.0 ± 10.0*	0.34 ± 0.03*	0.95 ± 0.11*	86.3 ± 5.1

**TABLE 4:** Performance measures from 5-choice serial reaction time task: Standard conditions of 0.75 s SD, 5 s ITI, 100 trials. Psilocybin “low” and “high” performers (N = 8 per subgroup).

Subgroup	Treatment	#Correct trials	#Incorrect trials	#Missed trials	%Correct	%Hit	PREM	PSV	Correct latency (s)	Magazine latency (s)	Total trials
Low performers	Vehicle	53.5 ± 6.2	15.8 ± 1.9	18.8 ± 5.8	76.9 ± 2.1	60.6 ± 5.5	5.6 ± 1.5	29.3 ± 2.7	0.83 ± 0.07	3.16 ± 0.44	88.0 ± 5.1
	Psilocybin (0.05 mg/kg)	62.4 ± 7.6*	13.0 ± 2.5	14.4 ± 5.4	81.8 ± 3.2*	69.1 ± 5.3*	6.5 ± 1.6	30.3 ± 6.5	0.72 ± 0.04*	4.77 ± 1.88	89.8 ± 7.7
	Psilocybin (0.1 mg/kg)	61.9 ± 5.1*	13.5 ± 2.1	14.3 ± 4.9	82.5 ± 1.9*	70.3 ± 4.6*	8.3 ± 2.7	35.1 ± 7.9	0.76 ± 0.05*	6.51 ± 2.66	89.6 ± 6.9
High performers	Vehicle	82.4 ± 3.0	6.4 ± 0.4	11.3 ± 3.0	92.8 ± 0.5	82.4 ± 3.0	5.0 ± 1.7	12.5 ± 2.0	0.63 ± 0.03	2.18 ± 0.18	100.0 ± 0.0
	Psilocybin (0.05 mg/kg)	81.9 ± 2.4	7.4 ± 1.8	10.8 ± 1.8	91.8 ± 1.9	81.9 ± 2.4	5.0 ± 1.8	14.3 ± 2.5	0.64 ± 0.03	2.37 ± 0.18	100.0 ± 0.0
	Psilocybin (0.1 mg/kg)	78.6 ± 4.7	6.8 ± 1.9	12.6 ± 2.7	91.9 ± 2.2	79.9 ± 3.9	3.3 ± 1.4	16.3 ± 4.7	0.66 ± 0.04	2.53 ± 0.22	98.0 ± 1.6

At the completion of experiments all rats were tested under an extended 5 s SD 5-choice test schedule. There were no performance differences in accuracy (% correct) between the “low” and “high” performer subgroups (data not shown).

##### Extended ITI Test Condition (10 s ITI, 0.3 s SD)

Extending the ITI from 5 s to 10 s, and reducing the SD from 0.75 s to 0.3 s resulted in a decrease in accuracy (% correct: 5 s ITI: 87.1 ± 1.5; 10 s ITI: 65.3 ± 2.5; *p* < 0.01) and an increase in PREM (5 s ITI: 10.1 ± 1.8, 10 s ITI: 45.4 ± 6.7; *p* < 0.01) and PSV (5 s ITI: 29.4 + 3.0, 10 s ITI: 43.2 ± 5.2; *p* < 0.01) responses consistent with this test format being specifically designed to challenge attention and response control.

**FIGURE 4:**
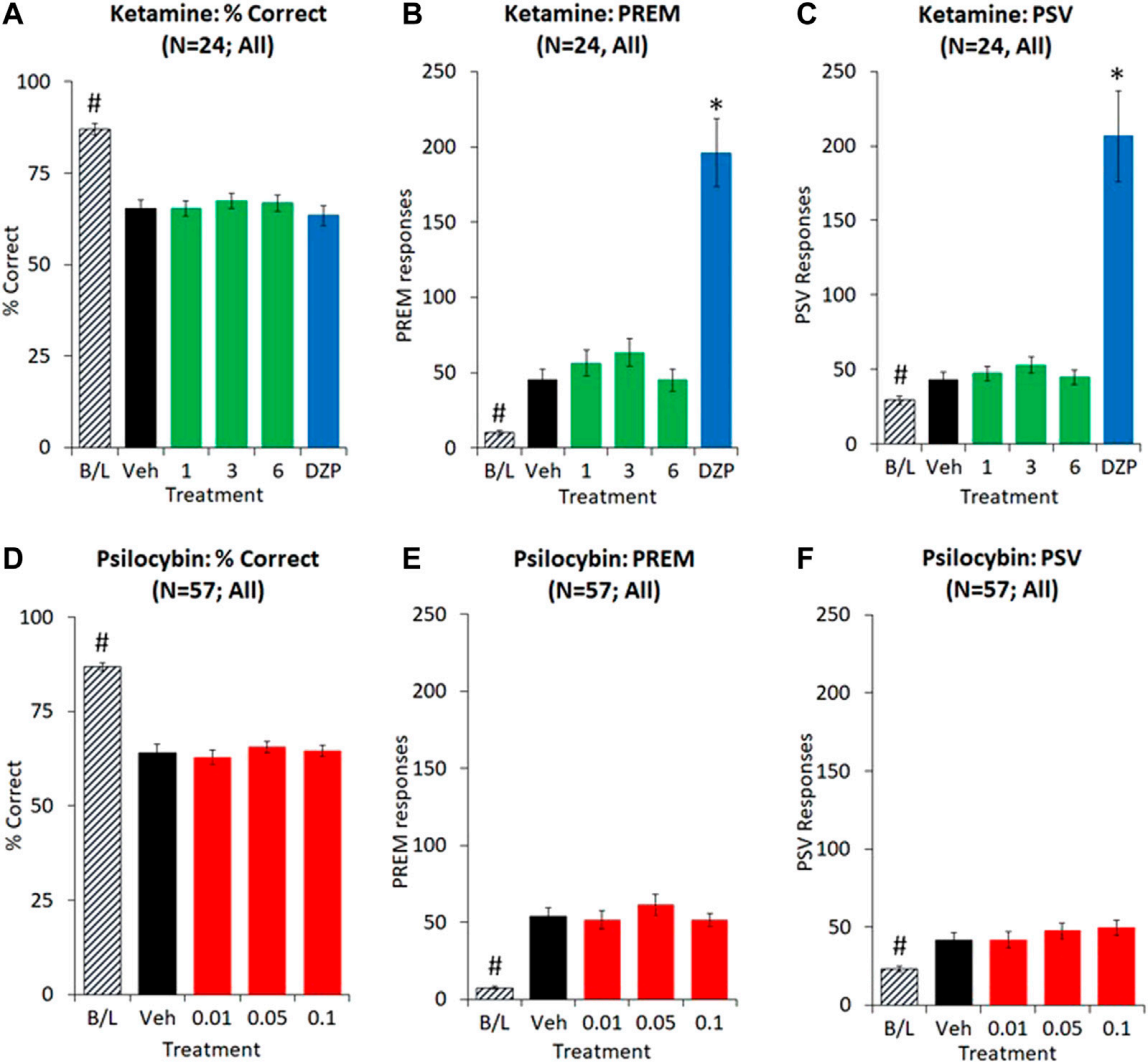
Characterisation of ketamine (1–6 mg/kg IP) and psilocybin (0.01–0.1 mg/kg SC) on performance of rats in the 5-choice serial reaction time task. Test conditions of 0.3 s SD, 10 s ITI, 100 trials; i.e. extended ITI. Data is presented for both drugs as % correct, **(A and D)**, total number of premature (PREM) responses **(B and E)**, and total number of perseverative (PSV) responses **(C and F)**. Dizocilpine (DZP, 0.02 mg/kg SC) was included as a reference comparator in the ketamine experiment. Ketamine experiment: N = 24; Psilocybin experiment: N = 57. *p < 0.05 vs. vehicle control, #*p* < 0.05 vs. all treatment groups (Dunnetts test following significant ANOVA).

Ketamine was tested at 1–6 mg/kg in a total of 24 rats, and dizocilpine (0.02 mg/kg) was also included as a reference control. Main effects of treatment on PREM (F4,92 = 37.3, *p* < 0.01; ƞ_p_^2^ = 0.62) and PSV (F4,92 = 26.9, *p* < 0.01; ƞ_p_^2^ = 0.54) essentially reflected a four-fold increase in both measures following dizocilpine (0.02 mg/kg) pretreatment (e.g. PREM: Veh: 45.4 ± 6.7, DZP: 196.1 ± 22.5), by contrast ketamine produced only a very modest effect (e.g. PREM: Veh: 45.4 ± 6.7, KET 3 mg/kg: 63.4 ± 9.4, *p* = 0.05). No main effect of treatment was recorded on other measures, e.g. % correct (F4,92 = 1.2, NS; ƞ_p_^2^ = 0.05), total trials (F4,92 = 2.3, *p* = 0.07; ƞ_p_^2^ = 0.09) (see Figures 4A–C and Table 5).

**TABLE 5:** Performance measures from 5-choice serial reaction time task: Test conditions of extended ITI, i.e. 0.3 s SD, 10 s ITI, 100 trials. Data is presented for all rats treated with ketamine (N = 24) or Psilocybin (N = 57). *p < 0.05 vs. vehicle (10 s ITI).

Treatment	# Correct trials	# Incorrect trials	# Missed trials	% Correct	% Hit	PREM	PSV	Correct lactency (S)	Magazine (S)	No.trials
Vehicle 5 s ITI	74.7 ± 2.7*	10.9 ± 1.3*	11.8 ± 1.8*	87.1 ± 1.5*	76.8 ± 2.3*	10.1 ± 1.8*	29.4 ± 3.0*	0.62 ± 0.02*	3.34 ± 0.39	97.4 ± 1.9
Vehicle 10 s ITI	53.8 ± 2.7	28.5 ± 2.2	15.3 ± 1.9	65.3 ± 2.5	55.1 ± 2.5	45.4 ± 6.7	43.2 ± 5.2	0.55 ± 0.02	5.76 ± 2.16	97.6 ± 2.1
Ketamine 1 mg/kg	54.3 ± 3.2	27.6 ± 1.6	13.6 ± 1.9	65.3 ± 2.1	56.1 ± 2.7	56.3 ± 8.8	47.4 ± 4.8	0.55 ± 0.02	4.91 ± 1.41	95.4 ± 2.8
Ketamine 3 mg/kg	58.6 ± 2.2	28.0 ± 1.7	12.6 ± 1.5	67.4 ± 2.0	58.7 ± 2.2	63.4 ± 9.4*	52.9 ± 5.5	0.51 ± 0.01	2.33 ± 0.13	99.2 ± 0.6
Ketamine 6 mg/kg	50.0 ± 4.1	22.7 ± 2.0	18.3 ± 3.5	66.8 ± 2.2	53.0 ± 3.7	45.0 ± 7.2	44.7 ± 4.7	0.55 ± 0.03	3.48 ± 0.97	91.0 ± 4.5
Dizocilpine 0.02 mg/kg	52.0 ± 4.2	27.5 ± 2.0	10.6 ± 1.4	63.4 ± 2.7	55.4 ± 3.4	196.1 ± 22.5*	206.5 ± 30.4*	0.53 ± 0.03	1.96 ± 0.13*	9.01 ± 3.6
Vehicle 5 s ITI	73.0 ± 1.9*	10.7 ± 0.9*	12.6 ± 1.2	86.9 ± 1.0*	75.5 ± 1.6*	7.4 ± 0.9*	23.2 ± 1.6*	0.67 ± 0.02*	3.63 ± 0.34	96.4 ± 1.2*
Vehicle 10 s ITI	49.9 ± 2.7	26.3 ± 1.6	11.9 ± 1.3	64.2 ± 2.1	54.8 ± 2.2	53.9 ± 5.9	41.5 ± 4.8	0.56 ± 0.03	3.76 ± 0.72	88.2 ± 3.5
Psilocybin 0.01 mg/kg	46.4 ± 3.0	24.8 ± 1.7	11.6 ± 1.2	62.9 ± 1.9	52.1 ± 2.3	51.6 ± 5.8	41.8 ± 5.3	0.60 ± 0.03	6.76 ± 3.00	82.8 ± 4.0
Psilocybin 0.05 mg/kg	50.1 ± 2.6	26.2 ± 1.5	10.6 ± 1.0	65.5 ± 1.5	56.6 ± 2.0	61.3 ± 6.8	47.5 ± 5.0	0.56 ± 0.02	5.53 ± 2.12	86.9 ± 3.6
Psilocybin 0.1 mg/kg	48.1 ± 2.8	25.6 ± 1.6	12.1 ± 1.1	64.6 ± 1.5	54.7 ± 2.0	51.5 ± 4.2	49.5 ± 4.7	0.58 ± 0.02	5.39 ± 2.18	85.8 ± 3.5

**FIGURE 5:**
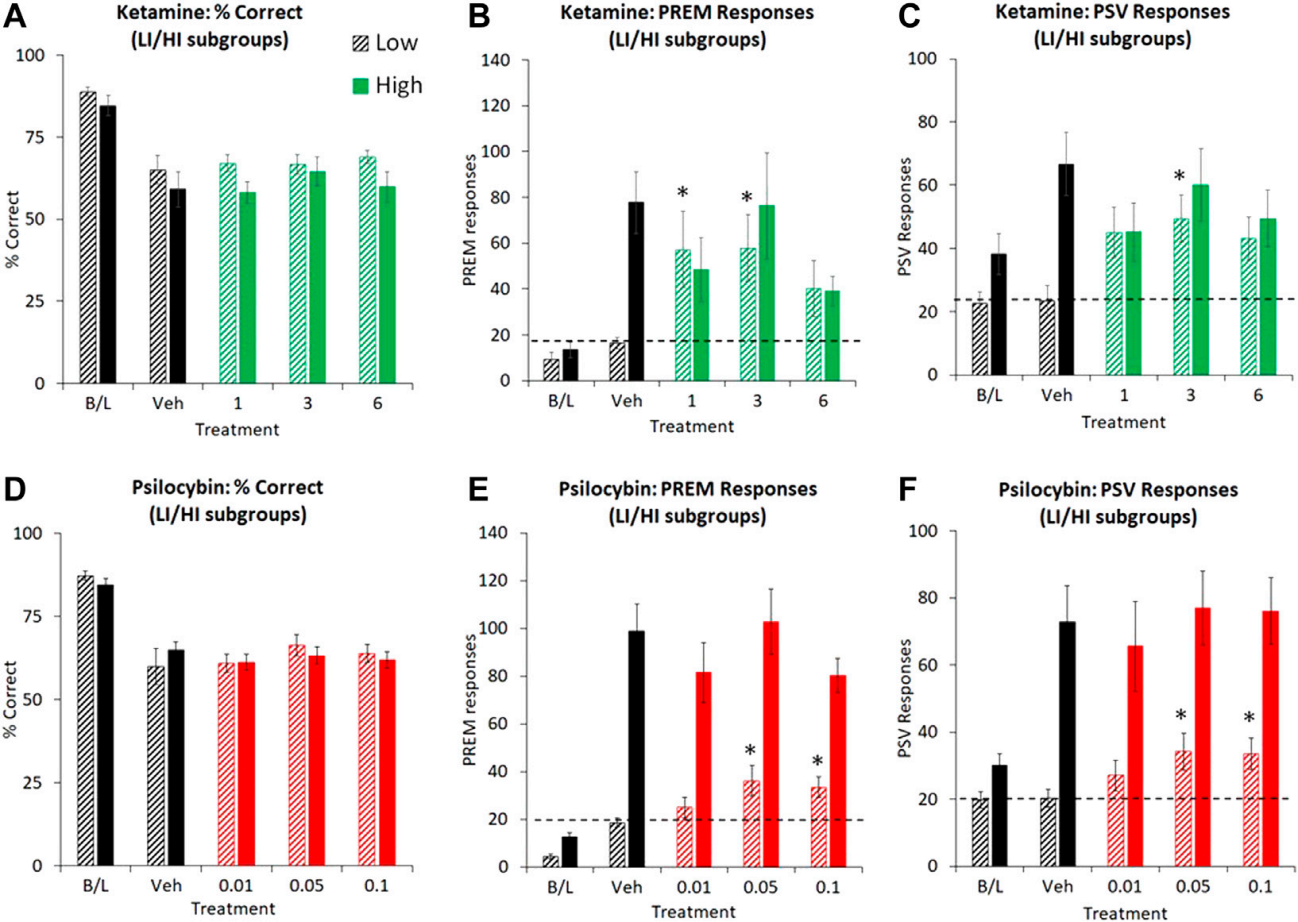
Characterisation of ketamine (1–6 mg/kg IP) and psilocybin (0.01–0.1 mg/kg SC) on performance of rats in the 5-choice serial reaction time task. Test conditions of 0.3 s SD, 10 s ITI, 100 trials; i.e. extended ITI. Data is presented for both drugs as % correct, **(A and D)**, total number of premature (PREM) responses **(B and E)**, and total number of perseverative (PSV) responses **(C and F)**. for subjects characterized as “low impulsive” (LI) or “high impulsive” (HI) performers (ketamine: N = 8 per tertile; psilocybin: N = 19 per tertile). The hashed line is to highlight the level of the “low impulsive” subgroup following vehicle pretreatment. Dizocilpine (DZP, 0.02 mg/kg SC) data were not included in these figures due to magnitude of change obscuring effects of ketamine and psilocybin. *p < 0.05 vs. vehicle control, #*p* < 0.05 vs. all treatment groups (Dunnetts test following significant ANOVA).

Subgrouping into LI and HI groups (N = 8 per subgroup) identified the effect of ketamine on PREM and PSV responses to be related to subgroup. Excluding dizocilpine from the analysis revealed a significant treatment × subgroup interaction for both PREM (F3,42 = 4.2; *p* = 0.01; ƞ_p_^2^ = 0.23) and PSV (F3,42 = 4.1; *p* = 0.01; ƞ_p_^2^ = 0.23) responses. Specifically, ketamine (1–3 mg/kg) increased PREM responses in the LI group, and tended to reduce this measure in the HI group. No other measures showed a significant treatment × subgroup interaction (see Figures 5A–C and Table 6).

**TABLE 6:** Performance measures from 5-choice serial reaction time task: Test conditions of extended ITI, i.e. 0.3 s SD, 10 s ITI, 100 trials. Ketamine “low” and “high” performers (N = 8 per subgroup).

Subgroup	Treatment	#Correct trials	#Incorrect trials	#Missed trials	%Correct	% Hit	PREM	PSV	Corect latency (s)	Magazine latency (s)	No trials
Low performers	Vehicle 5 s ITI	77.4 ± 3.3	9.5 ± 1.0	11.4 ± 2.8	88.9 ± 1.4	78.7 ± 2.9	9.1 ± 3.5	24.9 ± 3.6	0.60 ± 0.02	3.44 ± 0.39	98.4 ± 1.6
	Vehicle 10 s ITI	49.8 ± 7.6	24.5 ± 4.7	15.1 ± 2.4	65.0 ± 4.4	51.2 ± 6.4	16.6 ± 2.5	23.4 ± 4.9	0.51 ± 0.02	16.81 ± 13.99	89.4 ± 10.6
	Ketamine 1mg/kg	58.8 ± 3.8	28.4 ± 2.2	12.9 ± 3.4	67.1 ± 2.7	58.8 ± 3.8	57.1 ± 16.7	45.1 ± 8.0	0.51 ± 0.002	2.03 ± 0.12	100.0 ± 0.0
	Ketamine 3mg/kg	59.1 ± 2.8	29.4 ± 2.7	11.5 ± 1.8	66.8 ± 3.0	59.1 ± 2.8	57.9 ± 14.6	49.5 ± 7.4	0.50 ± 0.02	2.49 ± 0.26	100.0 ± 0.0
	Ketamine 6mg/kg	60.1 ± 2.8	27.0 ± 1.7	12.9 ± 2.7	68.9 ± 2.0	60.1 ± 2.8	40.1 ± 12.3	43.1 ± 6.8	0.52 ± 0.03	2.29 ± 0.03	100.0 ± 0.0
	Dizocilpine 0.02mg/kg	56.6 ± 4.2	30.9 ± 2.3	8.8 ± 1.9	64.3 ± 2.9	58.5 ± 3.4	174.5 ± 45.1	146.3 ± 26.3	0.49 ± 0.02	2.21 ± 0.29	96.3 ± 2.5
High performers	Vehicle 5 s ITI	72.7 ± 5.2	13.6 ± 3.1	8.3 ± 2.1	84.6 ± 3.1	77.2 ± 3.6	13.4 ± 3.5	38.2 ± 6.6	0.62 ± 0.03	2.90 ± 0.32	94.5 ± 5.5
	Vehicle 10 s ITI	48.1 ± 5.6	33.6 ± 5.4	11.9 ± 3.2	59.1 ± 5.3	51.9 ± 5.0	77.8 ± 13.5	66.6 ± 10.0	0.59 ± 0.06	2.50 ± 0.32	93.6 ± 6.4
	Ketamine 1 mg/kg	42.1 ± 6.1	29.3 ± 3.6	16.8 ± 3.9	58.2 ± 3.3	46.3 ± 4.4	48.4 ± 13.9	45.1 ± 9.2	0.57 ± 0.04	6.66 ± 3.45	88.1 ± 7.8
	Ketamine 3 mg/kg	53.9 ± 4.3	29.4 ± 4.0	15.3 ± 3.6	64.6 ± 4.3	54.1 ± 4.3	76.3 ± 23.3	60.1 ± 11.4	0.52 ± 0.03	2.19 ± 0.11	98.6 ± 1.4
	Ketamine 6 mg/kg	36.0 ± 8.5	20.8 ± 5.1	19.9 ± 8.2	59.9 ± 4.6	42.9 ± 7.9	39.0 ± 6.5	49.5 ± 8.9	0.59 ± 0.06	2.49 ± 0.58	76 ± 12.0
	Dizocilpine 0.02 mg/kg	53.0 ± 7.7	29.5 ± 4.4	9.6 ± 2.7	62.0 ± 6.0	55.9 ± 6.9	210.9 ± 29.6	268.3 ± 68.0	0.47 ± 0.04	1.98 ± 0.18	92.1 ± 4.9

#### Psilocybin

##### Standard Conditions (5 s ITI, 0.75 s SD)

Psilocybin was tested at 0.05–0.1 mg/kg in a total of 24 rats. No main effects of treatment were recorded on any performance measures (see Table 2 for summary). Subgrouping the rats into “low” and “high” performers based on % correct confirmed no main effects of treatment on any performance measure. A borderline interaction of treatment x subgroup was recorded on the accuracy measures of % correct (F2,28 = 2.2, *p* = 0.1; ƞ_p_^2^ = 0.13) and % hit (F2,28 = 3.0, *p* = 0.07; ƞ_p_^2^ = 0.17), and latency to make a correct response (F2,28 = 2.4. *p* = 0.1; ƞ_p_^2^ = 0.15). A sub-analysis conducted on the low performing cohort only (N = 8) revealed main effects of treatment on % hit (F2,14 = 4.8, *p* = 0.02; ƞ_p_^2^ = 0.41), but not % correct (F2,14 = 2.2, *p* = 0.1, NS; ƞ_p_^2^ = 0.24), or correct latency (F2,14 = 3.0, *p* = 0.08, NS; ƞ_p_^2^ = 0.30). This reflected a marginal improvement in both accuracy and response speed at both doses of psilocybin compared to vehicle pretreatment, however only % hit measure reached significance (Figures 3D–F and Table 4).

##### Extended ITI Test Condition (10 s ITI, 0.3 s SD)

Extending the ITI from 5 s to 10 s, and reducing the SD from 0.75 s to 0.3 s resulted in a decrease in accuracy (% correct: 5 s ITI: 86.9 ± 1.0; 10 s ITI: 64.2 ± 2.1; *p* < 0.01) and an increase in PREM (5 s ITI: 7.4 ± 0.9, 10 s ITI: 53.9 ± 5.9; *p* < 0.01) and PSV (5 s ITI: 23.2 ± 1.6, 10 s ITI: 41.5 ± 4.8; *p* < 0.01) responses.

Psilocybin was tested at doses of 0.01, 0.05 and 0.1 mg/kg in 57 rats. In this experiment there was a marginal trend for psilocybin to increase PREM and PSV responses (e.g. PREM: Veh: 53.9 ± 5.9; Psilo 0.05 mg/kg: 61.3 ± 6.8; *p* = 0.08 and PSV: Veh: 41.5 ± 4.8; Psilo 0.1 mg/kg: 49.5 ± 4.7; *p* < 0.01). There was no effect of psilocybin on accuracy (e.g. % correct: Veh: 64.2 ± 2.1, Psilo 0.05 mg/kg: 65.5 ± 1.5, Psilo 0.1 mg/kg: 64.6 ± 1.5) or on any other measures (see Figures 4D–F and Table 5).

Subgrouping into HI and LI tertiles (N = 19 rats per tertile) identified a borderline treatment × subgroup interaction on PREM (F3,108 = 2.3; *p* = 0.08; ƞ_p_^2^ = 0.06) but not PSV (F3,108 = 0.5, NS; ƞ_p_^2^ = 0.01) responses. Analysis restricted to the LI group (N = 19) revealed main effects of treatment on both PREM (F3,54 = 3.4; *p* = 0.02; ƞ_p_^2^ = 0.16) and PSV responses (F3,54 = 3.0; *p* = 0.04; ƞ_p_^2^ = 0.14), reflecting that psilocybin significantly increased PREM and PSV responses at the 0.05–0.1 mg/kg doses relative to vehicle pretreatment. Equivalent measures in the HI cohort were largely unaffected by psilocybin pretreatment. The LI and HI rats had equivalent levels of attentional accuracy (% correct: LI: 49.8 ± 7.6, HI: 48.1 ± 5.6, NS) which was unaffected by psilocybin (see Figures 5D–F and Table 7).

**TABLE 7:** Performance measures from 5-choice serial reaction time task: Test conditions of extended ITI, i.e. 0.3 s SD, 10 s ITI, 100 trials. Psilocybin “low” and “high” performers (N = 19 per subgroup).

Subgroup	Treatment	# Correct trails	# Incorrect trails	# missed trails	% Correct	%hit	PREM	PSV	Correct latency (s)	Magazine latency (s)	No. trails
Low performers	Vehicle 5 s ITI	68.5 ± 3.1	9.8 ± 1.1	13.3 ± 1.7	87.3±1.3	74.5 ± 2.1	4.4 ± 1.0	19.8 ± 2.5	0.72 ± 0.03	4.82 ± 0.83	91.7 ± 3.3
	Vehicle 10 s ITI	38.6 ± 5.4	23.2 ± 3.4	13.8 ± 2.4	59.8 ± 5.4	47.3 ± 4.6	18.6 ± 1.9	20.2 ± 2.7	0.58 ± 0.07	4.46 ± 1.64	75.6 ± 8.9
	Psllocybin 0.01 mg/kg	42.3 ± 5.6	24.4 ± 3.0	8.7 ± 1.5	60.9 ± 2.6	52.9 ± 2.8	24.9 ± 4.5	27.1 ± 4.5	0.69 ± 0.08	4.32 ± 1.29	75.5 ± 8.6
	Psllocybin 0.05 mg/kg	43.8 ± 5.4	22.6 ± 2.9	9.1 ± 1.3	66.3 ± 3.2	58.5 ± 3.5	36.2 ± 6.4	34.2 ± 5.5	0.58 ± 0.03	4.83 ± 1.53	75.5 ± 8.4
	Psllocybin 0.1 mg/kg	41.3 ± 5.4	23.2 ± 3.1	11.6 ± 2.3	64.0 ± 2.7	54.8 ± 3.5	33.5 ± 4.2	33.5 ± 4.7	0.59 ± 0.02	3.86 ± 1.33	76.0 ± 8.1
High performers	Vehicle 5 s ITI	72.9 ± 3.3	13.2 ± 1.7	12.5 ± 2.4	84.5 ± 1.9	73.7 ± 3.1	12.7 ± 1.8	30.1 ± 3.4	0.65 ± 0.03	3.12 ± 0.45	98.5 ± 1.0
	Vehicle 10 s ITI	52.9 ± 3.6	29.3 ± 2.7	11.5 ± 2.5	64.9 ± 2.5	56.8 ± 2.9	99.0 ± 11.3	72.8 ± 10.8	0.57 ± 0.03	4.20 ± 1.42	93.7 ± 4.0
	Psllocybin 0.01mg/kg	47.7 ± 4.3	28.9 ± 2.8	13.6 ± 2.1	61.2 ± 2.4	50.7 ± 3.3	81.5 ± 12.5	65.6 ± 13.3	0.62 ± 0.03	3.65 ± 0.89	90.3 ± 4.9
	Psllocybin 0.05mg/kg	51.0 ± 4.3	29.4 ± 2.9	10.8 ± 2.1	63.2 ± 2.6	52.8 ± 4.0	102.8 ± 13.6	77.1 ± 11.0	0.56 ± 0.04	2.54 ± 0.26	91.2 ± 5.3
	Psllocybin 0.1mg/kg	50.8 ± 3.6	31.5 ± 2.8	12.1 v 1.9	61.9 ± 2.5	52.9 ± 2.8	80.2 ± 7.1	76.1 ± 9.9	0.56 ± 0.03	3.18 ± 0.60	94.4 ± 3.2

### Pharmacokinetic Analysis of Ketamine and Psilocybin/Psilocin

#### Ketamine

Ketamine doses of 0.3, 3 and 30 mg/kg were selected. At 0.3–3 mg/kg doses, corresponding to efficacious doses in the progressive ratio and 5-CSRTT experiments, C_max_ was measured at ∼10 ng/ml (0.3 mg/kg) and ∼73 ng/ml (3 mg/kg). The 30 mg/kg dose which resulted in stereotypy and hyperlocomotion corresponded to ketamine plasma C_max_ of ∼820 ng/ml. The calculated PK parameters for ketamine are summarized in Figure 1C and Table 8.

**TABLE 8:** Calculated pharmacokinetic measures for ketamine.

	Ketamine dose (mg/kg) (mean ± SD)		
	0.3 mg/kg	3 mg/kg	30 mg/kg
t_max_ (h)	0.25 ± 0.0	0.25 ± 0.0	0.25 ± 0.0
c_max_ (ng/mL)	9.9 ± 0.8	73.2 ± 11.7	820 ± 330
Apparent t_1/2_(h)	0.52 ± 0.4	0.79 ± 0.05	0.73 ± 0.01
AUC_0-tlast_ (h * ng/mL)	8.3 ± 1.7	59.3 ± 11.7	607.0 ± 266
AUC_0-inf_ (h * ng/mL)	7.3 ± 1.2	63.3 ± 12.7	641 ± 281
MRT_0-inf_ (h)	0.80 ± 0.07	1.10 ± 0.08	1.00 ± 0.10

#### Psilocybin

Psilocybin doses of 0.05, 0.1, 1, and 10 mg/kg were selected for the determination of plasma concentrations of psilocybin and its primary metabolite psilocin. The 0.05 and 0.1 mg/kg doses corresponded to efficacious doses in the progressive ratio and 5-CSRTT experiments and resulted in plasma psilocin concentrations of 7–12 ng/ml. The 1 and 10 mg/kg doses corresponded to doses that induced overt behaviors (WDS/BMC) considered as 5-HT2A receptor mediated. At C_max_ these plasma psilocin levels were ∼83 ng/ml (1 mg/kg) and ∼1,100 ng/ml (10 mg/kg). Plasma exposure of psilocybin and psilocin determined as C_max_ or AUC increased with dose, although psilocin plasma levels showed higher inter-animal consistency. The calculated PK parameters for psilocybin are summarized in Table 9, and psilocin summarized in Figure 1F and Table 10.

**TABLE 9:** Calculated pharmacokinetic measures for psilocybin.

	Psilocybin dose (mg/kg) (mean ± SD)			
	0.05 mg/kg	0.1 mg/kg	1 mg/kg	10 mg/kg
$t_{\max}$ (h)	0.6 ± 0.2	0.32±0.05	0.36 ± 0.11	0.5 ± 0.9
$C_{\max}$ (ng/mL)	18.9 ± 9.4	31.4±15.1	78.3 ± 15.0	3565 ± 1762
$\text{Apparent}\;t_{1/2}$ (h)	n/a	n/a	0.45 ± 0.1	0.57 ± 0.1
${\text{AUC}}_{0-t\;\text{last}}$ (h*ng/mL)	10.7 ± 3.2	11.4±4.5	53.1 ± 8.9	1707 ± 884
${\text{AUC}}_{0-\inf}$ (h*ng/mL)	n/a	n/a	47.9 ± 10.2	2156 ± 1209
${\text{MRT}}_{0-\inf}\left(\text{h}\right)$	n/a	n/a	0.75 ± 0.10	0.72 ± 0.1

**TABLE 10:** Calculated pharmacokinetic measures for psilocin.

	Psilocybin dose (mg/kg) (mean ± SD)			
	0.5 mg/kg	0.1 mg/kg	1 mg/kg	10 mg/kg
t_max_ (h)	0.6 ± 0.4	0.54 ± 0.3	0.57 ± 0.3	0.71 ± 0.3
C_max_ (ng/mL)	7.14 ± 4.0	11.7 ± 6.8	83.3 ± 14.4	1106 ± 434
Apparent t_1/2_ (h)	1.00±0.20	0.92 ± 0.20	0.88 ± 0.10	1.70 ± 0.30
AUC_0-tlast_ (h*ng/mL)	10.1 ± 4.0	12.5 ± 6.4	117.0 ± 45.2	2280 ± 1524
AUC_0-inf_ (h*ng/mL)	10.3 ± 3.9	15.7 ± 5.6	143.0 ± 20.4	3376 ± 1056
MRT_0-inf_ (h)	1.73 ± 0.20	1.42 ± 0.30	1.53 ± 0.10	2.70 ± 0.40

## Discussion

The present series of experiments were designed to evaluate the behavioral properties of low doses and plasma concentrations of ketamine and psilocybin in the rat, with a view to identifying behavioral effects that might be relevant to the antidepressant and other therapeutic potential of both drugs. One of the first challenges to this line of research is defining a low dose range of ketamine and psilocybin. The approach taken in this study was to establish doses and plasma exposures of each drug for stereotyped behaviors characteristic of each drug and its distinct pharmacological class. Since behavioral stereotypies are often considered as the preclinical proxy for their psychomimetic property (Hanks and Gonzalez-Maeso, 2013; Halberstadt and Geyer, 2018), we focused on doses just below threshold for their induction. Based on this criterion we identified ketamine and psilocybin doses (and plasma exposures) of 0.3–3 mg/kg (10–70 ng/ml) and 0.05–0.1 mg/kg (7–12 ng/ml [psilocin]) respectively for investigation.

Preclinical studies explicitly examining low (“micro”) doses of ketamine and psilocybin are beginning to appear in the literature (Horsley et al., 2018; Meinhardt et al., 2020), albeit without any demonstration of potential beneficial effects. One of the limitations to these studies is that antidepressant potential has been typically investigated using tests such as forced swim and elevated plus maze, which lack human equivalence. These tests also overlook the trend to deconstruct complex clinical disorders into endophenotypes that may be more amenable to preclinical study and translation across the preclinical-clinical spectrum (Day et al., 2008; Markou et al., 2009). A diagnosis of MDD includes symptoms of depressed mood, anhedonia, fatigue/loss of energy (anergia), cognitive deficits including diminished/slowed ability to think or concentrate and feelings of guilt, worthlessness and suicidal ideation (van Loo et al., 2012; American Psychiatric Association, 2013). Therefore endophenotypes related to depression include anhedonia (impaired reward function), amotivation (lack of motivation/purpose) and impaired cognitive function (Hasler et al., 2004; Atique-Ur-Rehman and Neill, 2019; Treadway and Zald, 2011) which we addressed through the progressive ratio and 5-choice tasks.

A further consideration in the design of these experiments was an expectation that any effect of ketamine and psilocybin at low plasma concentrations was likely to be subtle, and potentially variable across a sample study population (see Horsley et al., 2018; Cameron et al., 2019; Meinhardt et al., 2020). We therefore exploited the heterogeneous nature of the performance level of rat populations across tasks such as PR and 5-CSRTT. Rats may be categorized based on performance differences in progressive ratio breakpoint, and thus serve as models of high vs. low motivation (Randall et al., 2012; Randall et al., 2015). Similarly rats may be categorized according to attentional accuracy or impulsive action under specific challenge conditions, thus providing models of high vs. low attention or impulsivity (Blondeau and Dellu-Hagedorn, 2007; Jupp et al., 2013; Hayward et al., 2016; Higgins et al., 2020a; Higgins et al., 2020b). Consequently, rats showing low motivation and/or attention may represent models of specific depression-relevant endophenotypes (Hasler et al., 2004; Treadway and Zald, 2011; Atique-Ur-Rehman and Neill, 2019). We identified three important considerations to this approach of subgrouping. Firstly, a requirement to identify an enduring nature to any performance subgroup classification. Secondly to establish “poor” performance is not a consequence of factors such as ill health, and thirdly a requirement for large sample sizes to ensure that subgroups were adequately separated and powered (Button et al., 2013). To address the former challenge, high/low performance subgroups were allotted based on 5–10 days baseline performance. Control experiments were conducted on the PR and 5-choice study cohorts which confirmed “low performance” was not associated with ill health or sensorimotor deficit. To address the third challenge, and to ensure at least some separation between subgroups but having due consideration to the principal of the 3R’s (replacement, refinement, reduction), we adopted the extreme tertile groups.

Considered as a whole, i.e. without subgrouping, despite group sizes of N = 24–72, we failed to identify any positive effect of ketamine or psilocybin on motivation or attention over the tested dose range. The most robust finding was a trend for a decline in performance following the 6 mg/kg dose of ketamine, which indicated the early phase of the descending limb of a biphasic dose response. This was confirmed by parallel experiments identifying even greater performance decline at 10 mg/kg (data not shown, but see Gastambide et al., 2013; Benn and Robinson, 2014; Nikiforuk and Popik, 2014).

Subgrouping rats based on break point and number of lever presses for food made available under a PR schedule of reinforcement identified rats that consistently ceased responding early (“low” responders), leading to low break points. Interestingly these rats had similar body weights, free feeding measures and open field activity compared to their high responder counterparts, suggesting any differences were unrelated to general health status, neurological function or appetite. In these low performers, both psilocybin (0.05–0.1 mg/kg) and ketamine (1–3 mg/kg) increased break point suggesting an increase in task motivation. These findings suggest that low doses of ketamine may relieve certain clinical signs related to depression (Xu et al., 2016), and further suggest that the doses and plasma concentrations of ketamine and psilocybin as described in the present study may have utility in treating subtypes of mental illnesses characterized by amotivation and anhedonia in particular.

In the 5-CSRTT, the effects of ketamine and psilocybin were evaluated in two separate task schedules. In the first, rats were tested under standard conditions of 0.75 s SD, 5 s ITI. Segregation of rats into high and low performers based on accuracy (% correct), revealed a trend for both psilocybin and ketamine to increase accuracy at equivalent doses to those effective in the PR task. In the case of psilocybin, the more robust measure of efficacy was the % hit measure, which also accounts for errors of omission as well as commission (incorrect response). Speed of responding was also marginally increased further supporting a performance improvement.

The second 5-CSRTT experiment utilized conditions of extended ITI (5 s vs. 10 s) and reduced stimulus duration (0.75 s vs. 0.3 s). The principal challenge is to response control, lengthening the ITI from 5 s to 10 s produces a significant increase in both PREM and PSV responses, a consistent and widely reported finding (Robbins, 2002; Jupp et al., 2013; Barlow et al., 2018; Higgins et al., 2020a,b). Subgrouping rats, based on the level of PREM responses under the 10 s ITI schedule, into “Low” and “High” impulsives (LI vs. HI) highlights a wide range of responders typically seen under this schedule (Jupp et al., 2013; Fink et al., 2015; Barlow et al., 2018; Higgins et al., 2020a). Importantly there is a reasonable consistency of performance on this measure over repeated tests as demonstrated by the HI rats having higher PREM scores under the 5 s ITI, albeit at markedly lower levels. PSV responses are also higher in the HI cohort, consistent with the HI rats demonstrating a deficit in inhibitory response control.

Similar findings for both ketamine and psilocybin were noted in this test schedule. While neither drug affected accuracy (measured as % correct), either in all, or HI/LI classified rats; both increased PREM and PSV responses in the LI cohort, supporting an increase in impulsive action. It should be noted that the magnitude of change produced by both ketamine and psilocybin was relatively small (∼2-fold) and confined to the LI subgroup. Certainly, the magnitude of change contrasted sharply with the 4-fold increase noted in rats pretreated with dizocilpine under the same 10 s ITI schedule (see also Higgins et al., 2005; 2016; Benn and Robinson, 2014). Previous studies have also described increased PREM responses following pretreatment with the phenethylamine 5-HT2A agonist DOI (Koskinen et al., 2000; Koskinen and Sirvio, 2001; Blokland et al., 2005; Wischhof and Koch, 2012; Fink et al., 2015), typically at doses lower than those which induce signs of WDS/BMC (Fink et al., 2015; Halberstadt and Geyer, 2018).

Impulsivity is a construct that may be viewed in two forms: functional and dysfunctional (Dickman, 1990). Dysfunctional impulsivity is associated with psychiatric conditions such as substance abuse and OCD and thus carries a negative context. For example, associations between high impulsive trait and drug seeking behaviors have been reported both preclinically and clinically (Grant and Chamberlain, 2004; Jupp et al., 2013). Functional impulsivity has been described as a tendency to make quick decisions when beneficial to do so, and may be related to traits such as enthusiasm, adventurousness, activity, extraversion and narcissism. Individuals with a high functional impulsivity are also reported to have enhanced executive functioning overall (Dickman, 1990; Zadravec et al., 2005; Burnett Heyes et al., 2012). Viewed in this more positive context, the feature of psilocybin and ketamine to promote impulsive behavior selectively in a LI cohort may be relevant in supporting a potential to treat depression and other mental disorders.

One advantage of being able to study pharmacological effects at low doses in an experimental setting, is the ability to probe for an underlying neurobiological mechanism, which would serve to establish this pattern of use within a scientific framework. Presumably these doses result in a low level of target site occupancy, which in the case of psilocybin is the serotonin 5-HT2A receptor (Vollenweider et al., 1998; Tylš et al., 2014; Nichols, 2016; Kyzar et al., 2017). At higher doses and plasma exposure, and consequently higher levels of target occupancy, psychomimetic effects begin to emerge. In this respect, the recent study of Madsen et al., (2019) is of interest. These workers reported a correlation between the psychedelic effects of psilocybin (40–100% Likert scale maximum) and CNS 5-HT2A receptor occupancy (43–72%) and plasma psilocin levels (2–15 ng/ml). Increases in subjective intensity was correlated with both increases in 5-HT2A receptor occupancy and psilocin exposure. Based on these data, it is estimated that at 5-HT2A receptor occupancies up to ∼15%, no perceptual effects occur (Madsen and Knudsen, 2020).

5-HT2A receptors are widely distributed within cortical zones, notably layer II-V (Santana et al., 2004; Mengod et al., 2015), and also in subcortical regions such as the DA nigrostriatal and mesocorticolimbic pathways where they appear to positively regulate tone, at least under certain physiological conditions (Doherty and Pickel, 2000; Nocjar et al., 2002; Bortolozzi et al., 2005; Alex and Pehek, 2007; Howell and Cunningham, 2015; De Deurwaerdère, and Di Giovanni, 2017). One plausible hypothesis is that at low nanomolar plasma concentrations, psilocybin (or LSD, mescaline etc.) may preferentially target a subset of 5-HT2A receptors, possibly those localized to subcortical DA systems where activation has been reported to increase firing and tonicity of these pathways (Alex and Pehek, 2007; Howell and Cunningham, 2015; De Deurwaerdère, and Di Giovanni, 2017 for reviews). In turn this might be expected to promote behaviors related to motivation, attention and impulse control as noted in the PR and 5-choice experiments. Activation of cortical 5-HT2A receptors may account for the subjective/perceptual effects once a critical (higher) drug [plasma] threshold has been reached (Nichols, 2016; Kyzar et al., 2017; Madsen et al., 2019; Vollenweider and Preller, 2020).

In the case of ketamine, the relevant target is most likely the NMDA subtype glutamate receptor (Lodge and Mercier, 2015; Mathews et al., 2012; Corriger and Pickering, 2019; although note; Zanos et al., 2018), which is comprised of a tetrameric receptor complex composed of NR1 subunits, combined with NR2A-D subunits and, in some cases, NR3A-B subunits. The NR2A-D subunits exist in an anatomically distinct manner, with the NR2A and NR2B subunits predominant in forebrain; the NR1 subunit having a broader distribution being a constituent of all NMDA channels (Kew and Kemp, 2005; Traynelis et al., 2010). Potentially at low ketamine doses, there may be a preferential interaction between ketamine and specific NMDA channel subtypes (see Lodge and Mercier, 2015), and/or regional subpopulations which underlies the pharmacological effects of these doses of ketamine in preclinical and clinical contexts. We and others have reported on apparently pro-cognitive effects of non-competitive NMDA antagonists, typically dizocilpine, when tested at low doses (Mondadori et al., 1989; Jackson et al., 2004; Higgins et al., 2003; 2016; Guidi et al., 2015). A better understanding of the neurobiological mechanisms that underlie these effects may provide useful insight toward understanding the clinical benefit of low doses of ketamine in humans.

An interesting feature to emerge from this work was the similar profile of ketamine and psilocybin across the PR and 5-choice experiments. Both drugs increased break point in low performers, improved attention in low performer subgroups, and increased PREM/PSV responses in LI rats. Horsley et al., (2018) also reported a similar pattern of both drugs across various elevated plus maze measures, although the effects were suggestive of a mild anxiogenic profile. Despite their differing pharmacology, there is accumulating evidence from a variety of sources that the NMDA and 5-HT2A receptors are functionally intertwined. Vollenweider has highlighted the overlapping psychotic syndromes produced by serotonergic hallucinogens and psychotomimetic anesthetics associated with a marked activation of the prefrontal cortex and other overlapping changes in temporoparietal, striatal, and thalamic regions (Vollenweider, 2001; Vollenweider and Kometer, 2010) suggesting that both classes of drugs may act upon a common final pathway. Secondly, 5-HT2A receptor antagonists attenuate a variety of putative psychosis-related behaviors induced by NMDA channel block, including behavioral stereotypy and disrupted PPI (Varty and Higgins, 1995; Varty et al., 1999; Higgins et al., 2003), a property that likely contributes to the antipsychotic efficacy of atypical neuroleptics such as clozapine, risperidone (Meltzer, 1999; Remington, 2003). Furthermore, a cellular coexpression of 5-HT2A and NMDA receptors has been described in multiple brain regions, including VTA, striatum and cortex (Wang and Liang, 1998; Rodriguez et al., 1999; Rodriguez et al., 2000). Therefore, studying these drugs at the low dose range may also provide further insights into how these receptor systems may interact.

In conclusion, the present studies have characterized for the first time, a positive effect of ketamine (0.3–3 mg/kg [plasma] 10–70 ng/ml) and psilocybin (0.05–0.1 mg/kg [psilocin plasma] 7–12 ng/ml) on behaviors related to endophenotypes of amotivation and anhedonia. The overall effect sizes are modest, which might be expected at the doses and concentrations studied, where the degree of target occupancy is likely to be low and subject to individual differences in drug pharmacodynamics and pharmacokinetics. Each of these factors will impact on treatment response across a study population (Levy, 1998; Dorne, 2004). Limitations to the present study include a restriction to male test subjects, and on single acute doses. Future studies should extend to both male and female subjects, and alternative dosing schedules. Nonetheless, the studies are important in that they define a potentially efficacious dose and plasma exposure range and provide a framework for early safety studies and further scientific investigation into the neurobiology of these drugs in the low dose range.

## Data Availability

The raw data supporting the conclusions of this article will be made available by the authors, without undue reservation.
